# Supplementary material for: Gastric Enzyme Supplementation Inhibits Food Allergy in a BALB/c Mouse Model
Source: Nutrients. 2021 Feb 26;13(3):738. doi: 10.3390/nu13030738 (PMC7996948; doi:10.3390/nu13030738)
Supplement: Supplementary file 1 [file nutrients-13-00738-s001.pdf]

Table S1. Experimental procedure

Exp. 1	Gastric acid suppression	Gastric enzyme solution (GES)
	PPI	no GES
	PPI	500µg GES, 5 min before measurement
	PPI	1000µg GES, 5 min before measurement
	PPI	1000µg GES, immediately before measurement
	no PPI	no GES
**Exp. 2**	**Allergy induction**	**Oral challenge**
	OVA+PPI+Sucralan+GES	200µg OVA
	OVA+PPI+Sucralan	200µg OVA
	Ip injection	200µg OVA
	naive	-
**Exp. 3**	**Allergy induction**	**Oral challenge**
	OVA+PPI+Sucralan	1000µg GES, 5min before OVA challenge
	OVA+PPI+Sucralan	1000µg GES, immediately before OVA challenge
	OVA+PPI+Sucralan	-

Figure S1

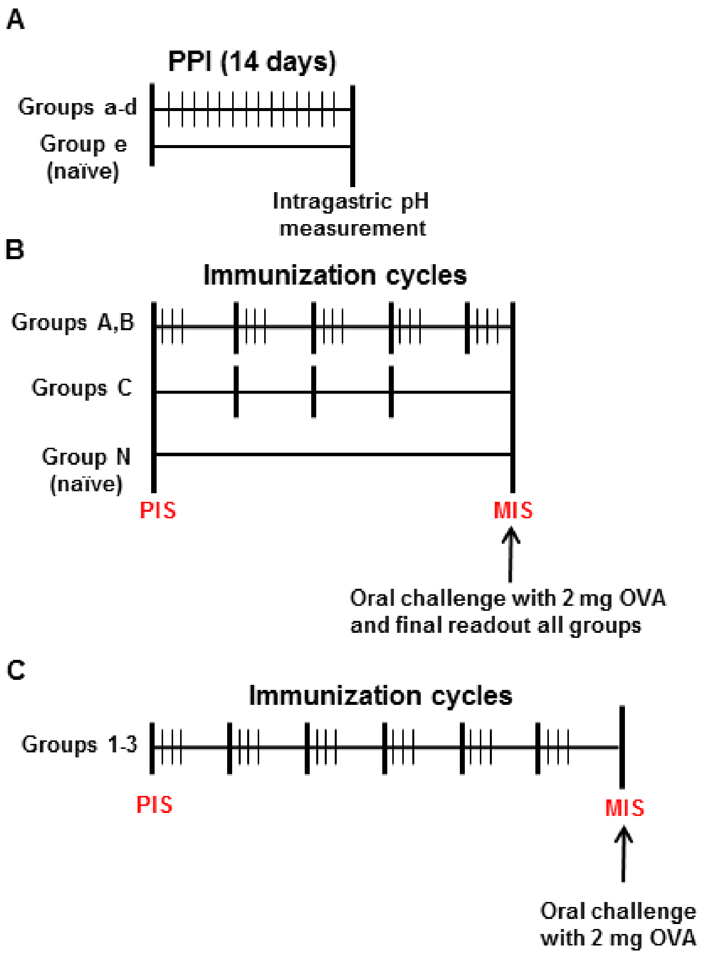

Figure S1. *Mouse treatment protocols of experiments 1-3.*

Figure S2

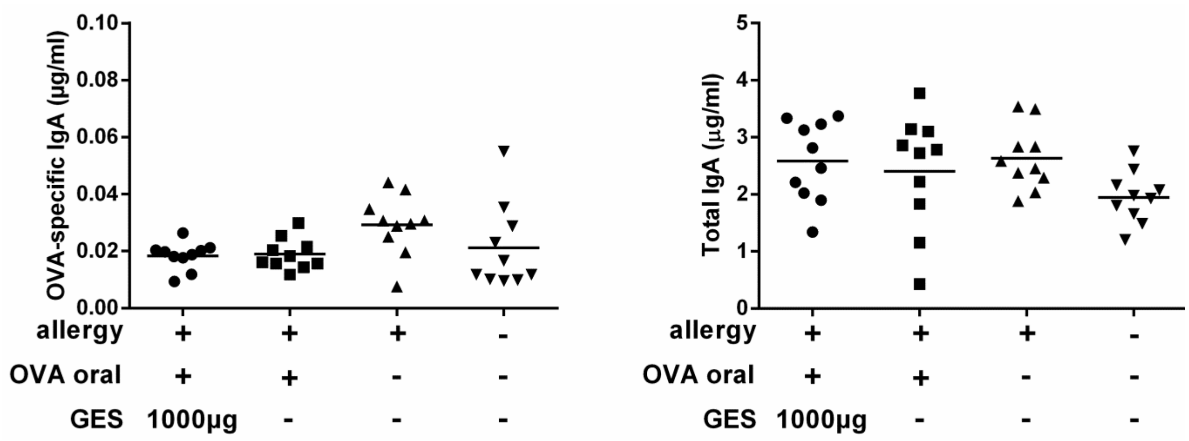

Figure S2. *Comparable OVA-specific IgA titers in intestinal lavages after allergy induction with different protocols.* Intestinal lavages were collected and evaluated for total and OVA-specific IgA by ELISA. No differences were observed regarding local IgA production between groups receiving oral OVA sensitizations under gastric acid suppression with and without GES supplementation, the positive controls after ip immunizations or the naïve animals.

Figure S3

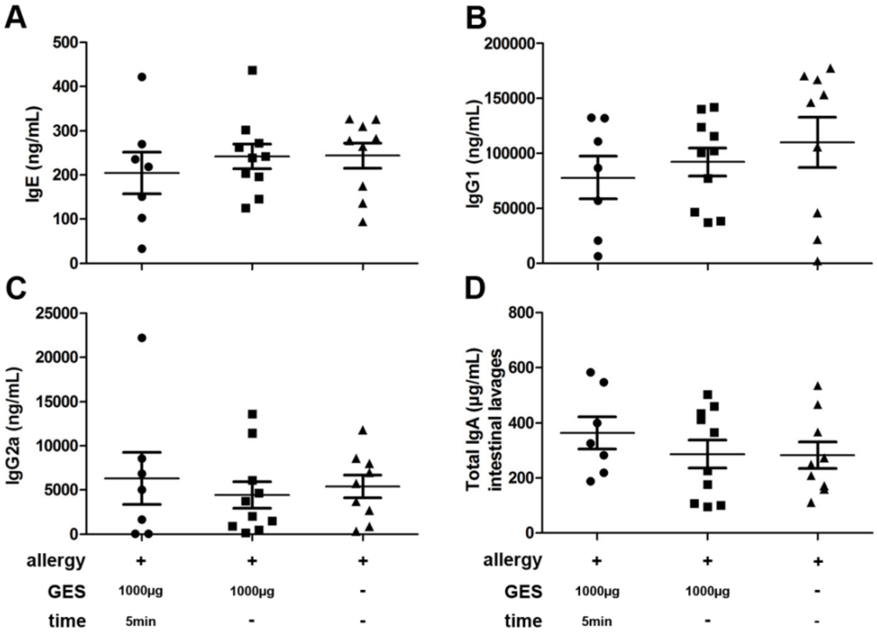

Figure S3. *Comparable titers of OVA-specific IgE, IgG1 and IgG2a and total IgA were found in serum in intestinal lavages of food allergic animals subjected to oral OVA challenge.* After 6 rounds of oral immunizations and a single oral challenge with OVA, blood was collected by cardiac puncture and intestinal lavages were harvested from all groups. Antibodies levels were measured by ELISA. OVA-specific IgE (**A**), OVA-specific IgG1 (**B**), OVA-specific IgG2a (**C**) and total IgA (**D**) showed comparable antibody titers between the 3 groups.
